# Predominant Distribution of the RNAi Machinery at Apical Adherens Junctions in Colonic Epithelia Is Disrupted in Cancer

**DOI:** 10.3390/ijms21072559

**Published:** 2020-04-07

**Authors:** Joyce Nair-Menon, Amanda C. Daulagala, Dean M. Connor, Lauren Rutledge, Trevor Penix, Mary Catherine Bridges, Bridgette Wellslager, Demetri D. Spyropoulos, Cynthia D. Timmers, Ann-Marie Broome, Antonis Kourtidis

**Affiliations:** 1 Department of Regenerative Medicine and Cell Biology, Medical University of South Carolina, 173 Ashley Avenue, Charleston, SC 29425, USA; menonnj@musc.edu (J.N.-M.); gunarath@musc.edu (A.C.D.); lauren.rutledge24@gmail.com (L.R.); tsp4471@rit.edu (T.P.); bridgmar@musc.edu (M.C.B.); wellslag@musc.edu (B.W.); 2 Department of Cell and Molecular Pharmacology and Experimental Therapeutics, Medical University of South Carolina, 173 Ashley Avenue, Charleston, SC 29425, USA; connord@musc.edu (D.M.C.); broomelab@gmail.com (A.-M.B.); 3 Department of Pathology and Laboratory Medicine, Medical University of South Carolina, 173 Ashley Avenue, Charleston, SC 29425, USA; spyropdd@musc.edu; 4 Department of Medicine, Medical University of South Carolina, 171 Ashley Avenue, Charleston, SC 29425, USA; timmers@musc.edu

**Keywords:** E-cadherin, p120 catenin, PLEKHA7, DROSHA, DGCR8, Ago2, GW182, RNA interference, colorectal, tumor suppressor

## Abstract

The RNA interference (RNAi) machinery is an essential component of the cell, regulating miRNA biogenesis and function. RNAi complexes were thought to localize either in the nucleus, such as the microprocessor, or in the cytoplasm, such as the RNA-induced silencing complex (RISC). We recently revealed that the core microprocessor components DROSHA and DGCR8, as well as the main components of RISC, including Ago2, also associate with the apical adherens junctions of well-differentiated cultured epithelial cells. Here, we demonstrate that the localization of the core RNAi components is specific and predominant at apical areas of cell-cell contact of human normal colon epithelial tissues and normal primary colon epithelial cells. Importantly, the apical junctional localization of RNAi proteins is disrupted or lost in human colon tumors and in poorly differentiated colon cancer cell lines, correlating with the dysregulation of the adherens junction component PLEKHA7. We show that the restoration of PLEKHA7 expression at adherens junctions of aggressively tumorigenic colon cancer cells restores the junctional localization of RNAi components and suppresses cancer cell growth in vitro and in vivo. In summary, this work identifies the apical junctional localization of the RNAi machinery as a key feature of the differentiated colonic epithelium, with a putative tumor suppressing function.

## 1. Introduction

Colorectal cancer is the third most prevalent and second deadliest form of cancer [1]. This implies that there are still gaps in our understanding of the disease [2]. A common feature of colorectal tumors is disruption and eventual loss of epithelial tissue architecture and integrity, which occurs in early or even pre-cancerous stages of the disease [3,4]. However, the extent that disruption of colonic epithelial architecture contributes to pro-tumorigenic cell transformation is still unclear [2,3,4,5].

The adherens junction is a cell-cell adhesion structure critical for organ development, tissue architecture, and cell function [6,7]. E-cadherin is the core adhesion molecule in epithelial cells and is stabilized through its association with p120 catenin (p120) [8]. Due to its essential function in epithelial homeostasis, E-cadherin has long been considered a tumor suppressor, which is downregulated during epithelial-mesenchymal transition, promoting tumor progression and metastasis. However, numerous studies in recent years have challenged this notion [5,9]. For example, we and others have shown that E-cadherin is still expressed in most tumors examined and that E-cadherin-p120 complexes are required for cancer cell survival, proliferation, anchorage-independent growth, as well as for collective cell migration [5,10,11,12,13,14,15,16,17,18]. These findings complicated our understanding of the function of E-cadherin complexes in tumorigenesis.

Although E-cadherin-based cell-cell contacts form across lateral membranes between adjacent cells, mature adherens junctions appear in well-differentiated epithelia and specifically localize at the apical areas of cell-cell contact, where they tether to circumferential actin to form the zonula adherens [6,7]. This and other findings point toward structural differences between the apical adherens junctions and basolateral areas of cell-cell contact. For example, it has been shown that a p120-binding partner called PLEKHA7 specifically localizes at the apical adherens junctions of well-differentiated epithelial cells and tissues, but not at the lateral areas of cell-cell contact [10,19,20]. In addition to these structural differences between the apical and basolateral complexes at areas of cell-cell contact, we recently showed that these complexes are also functionally distinct. In particular, we demonstrated that the apical adherens junctions can suppress pro-tumorigenic cell transformation through PLEKHA7, whereas the basolateral cell-cell junction complexes promote it, in the absence of PLEKHA7 [10,21,22]. In agreement with these findings, we and others have shown that PLEKHA7 is broadly mis-localized or downregulated in human breast and kidney tumors, although E-cadherin is still broadly expressed at areas of cell-cell contact [10,23]. These findings provided a mechanistic basis to explain why E-cadherin complexes can act both in a pro- and in an anti-tumorigenic fashion and revealed that other cadherin-junction components have to be taken into consideration, when assessing the role of E-cadherin in tumorigenesis.

Our investigation to understand how the adherens junctions may be acting as a tumor suppressor led to the revelation that PLEKHA7 recruits numerous RNA-binding proteins, including the main complexes of the RNA interference (RNAi) machinery [10,24,25]. More specifically, we showed that PLEKHA7 recruits the core components of the microprocessor complex, namely DROSHA and DGCR8, as well as the RNA-induced silencing complex (RISC), including its key enzymatic component Ago2 [10,25]. Notably, we showed that this interaction occurs specifically at the apical adherens junctions of well-differentiated epithelial cells, such as human colon Caco2 and canine kidney MDCK cells [10,25]. By recruiting these complexes, PLEKHA7 regulates the processing and activity of a number of tumor-suppressing miRNAs, such as miR-30b, miR-24, miR-200c, and miR-203a [10,25]. PLEKHA7 depletion in Caco2 cells results in the disruption of the localization of the RNAi machinery at the adherens junctions and in decreased levels and activity of these miRNAs, eventually promoting oncogene upregulation and pro-tumorigenic transformation of Caco2 cells [10,25]. These findings provided a path to explain the function of the apical adherens junction complex as a suppressor of pro-tumorigenic cell transformation. In addition, these results challenged the broadly accepted notion that the microprocessor complex localizes solely in the cell nucleus and that the RISC specifically localizes in the cytoplasm, by demonstrating their additional association with the apical adherens junctions of well-differentiated epithelial cells [24,26,27]. Since we obtained these data in cultured epithelial cells, such as colon Caco2 cells, the question that emerged is whether the junctional localization of the RNAi machinery is broad and physiologically relevant to other cells and tissues, either normal or diseased. In this work, we seek to examine whether this subcellular distribution of the microprocessor and RISC at apical adherens junctions is indeed a feature of the well-differentiated colonic epithelium. We also examine whether this localization is maintained or lost in colon cancer cells and tumors, since our findings point towards a putative tumor suppressing mechanism that is potentially disrupted in cancer. In addition, we test whether we can restore this junctional localization of RNAi in colon cancer cell lines and look at effects on their growth. Our findings presented here confirmed our predictions, indicating that the apical adherens junction-associated RNAi machinery is a predominant feature of well-differentiated colonic epithelia, which is broadly disrupted in colon cancer.

## 2. Results

### 2.1. The Core Components of the RNAi Machinery Localize at Apical Adherens Junctions in Normal Colon Epithelia

**Figure 1:**
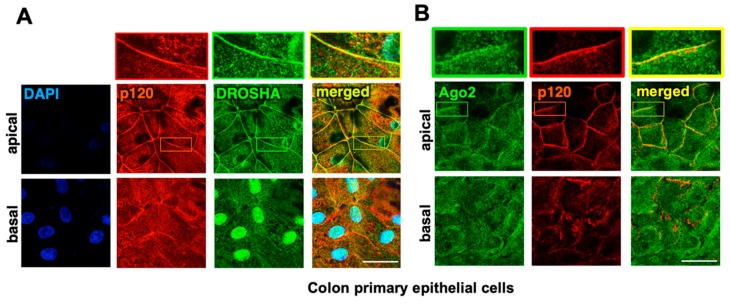
The core RNAi components DROSHA and Ago2 localize at apical adherens junctions. Primary colonic epithelial cells were stained by immunofluorescence for (**A**) DROSHA, (**B**) Ago2, and co-stained with p120; DAPI is the nuclear stain. Image stacks were acquired by confocal microscopy covering the entire cell monolayer between the basal and the apical areas of cell-cell contact. Representative apical-basal image stacks and merged composites are shown. Enlarged parts of images on top of each stack indicate areas of cell-cell contact. Scale bars: 20 μM.

We have recently shown that the adherens junctions recruit the core components of the RNAi machinery, such as DROSHA, DGCR8, and Ago2, through PLEKHA7 [10,25]. In those studies, we primarily used Caco2 colon intestinal epithelial cells. These cells are well-differentiated, they can fully polarize in culture and are broadly used as a versatile model of the fully differentiated colonic epithelium [28,29,30], which is why we used them as our model. Still, they are derived from a primary colon adenocarcinoma and cannot be considered normal. Therefore, to examine the relevance of our findings to the normal colonic epithelium, we obtained primary colonic epithelial cells. These cells do not proliferate and cannot be extensively used in culture; however, we were able to plate them on coverslips to perform immunofluorescence and image them using confocal microscopy. This approach confirmed that DROSHA and Ago2 localize at the adherens junctions of these cells, in agreement with the findings in Caco2 cells (Figure 1). Since these cells can also partially polarize under these culture conditions, we performed a fine optical dissection of the apical and the basolateral areas of cell-cell contact of these cells, using confocal microscopy. Notably, junctional localization of both DROSHA and Ago2 is restricted at the apical adherens junctions, whereas they were predominantly nuclear or cytoplasmic, respectively, at that basal areas of cell-cell contact of these cells. These results are in agreement with our previous findings in polarized Caco2 cells and further demonstrate the specific localization of RNAi complexes at apical adherens junctions.

**Figure 2:**
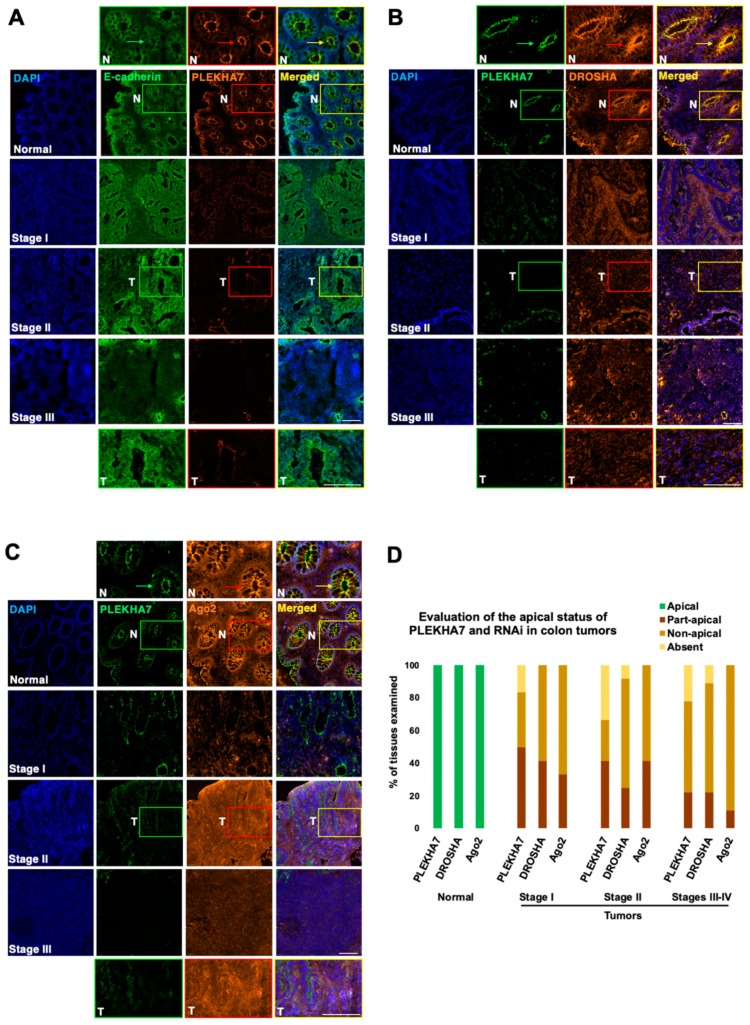
The core components of the RNAi machinery localize at apical adherens junctions of normal colon tissues but are absent from areas of cell-cell contact in colon tumors. Examination of a total of colon normal and matched tumor tissues from *n* = 33 patient tissue samples from stages I (*n* = 12), II (*n* = 12), III (*n* = 8) and IV (*n* = 1), to assess localization status of RNAi machinery components. (**A–C**) Immunofluorescence staining for E-cadherin, PLEKHA7, DROSHA, Ago2. DAPI is the nuclear stain. Representative tissues from normal tissues and from each stage are shown. Enlarged parts of images on top of each stack are from normal (N) tissues, whereas at the bottom are from tumor tissues (T). Arrows indicate apical localization. Scale bars: 100 μM. (**D**) Overall assessment of the apical junctional localization status of PLEKHA7, DROSHA, Ago2 in all *n* = 33 tissues examined; results show the percentile of tissues that each marker exhibits apical, partial-apical, no apical localization, or is absent.

To further examine the localization pattern of RNAi complexes in the colonic epithelium, we obtained colon tissues from colon cancer patients, as well as matched normal colon tissue samples. We performed immunofluorescence of these tissues for DROSHA, DGCR8, and Ago2, using E-cadherin as our cell-cell junction marker and PLEKHA7 as our specific apical adherens junctions marker, according to what previous studies have extensively demonstrated [10,19,20]. We assessed tissues from a total of 33 patients. As expected, in normal tissues, E-cadherin is junctional both at apical and lateral areas of cell-cell contact, whereas PLEKHA7 strictly localizes at the apical areas of cell-cell contact and also highlights the apical areas of colonic crypts, where the fully differentiated epithelial cells of crypts reside [5,31] (Figure 2A). Neither E-cadherin, nor PLEKHA7 stain the stromal tissue, but strictly the epithelial, confirming the specificity of our findings to the colonic epithelium.

Remarkably, localization of DROSHA, DGCR8, and Ago2 in all normal tissues we examined was primarily at the apical areas of cell-cell contact of the crypts of these tissues, co-localizing with PLEKHA7 (Figure 2B,C and Figure S1). Although nuclear localization of DROSHA and DGCR8 and cytoplasmic localization of Ago2 is also observed in these samples, their apical localization seems predominant and highlights the apical areas of colonic crypts. These observations are in agreement with our findings both in Caco2 cells and in primary colon epithelial cells (Figure 1) and demonstrate that the core components of the RNAi machinery primarily localize at the apical adherens junctions of well-differentiated human colonic epithelial tissues.

### 2.2. PLEKHA7 and RNAi Components Are Dysregulated in Human Colon Tumors

Our previous experimentation with Caco2 cells showed that PLEKHA7 depletion results in the loss of junctional localization of RNAi components [10,25]. We also introduced data from breast and kidney tumors showing extensive mis-localization or downregulation of PLEKHA7 [10]. However, we have not assessed the status of PLEKHA7 in colon tumors. In addition, we have not examined the status of RNAi complexes in any of these tissues. Therefore, here, we examined DROSHA, DGCR8, and Ago2 localization in the colon tumor tissues we collected, in comparison to their normal matched tissues discussed above. We used E-cadherin and PLEKHA7 as our lateral and apical cell-cell junction markers, as above. In agreement with our previous findings in breast and kidney tissues, we found that PLEKHA7 is extensively mis-localized in colon tumors from all stages (Figure 2A–C and Figure S2). More specifically, apical and/or junctional localization of PLEKHA7 appears to be either fragmented or the protein downregulated in colon tumors (Figure 2A–C and Figure S2). Interestingly, apical junctional localization of RNAi components follows the same pattern in these tumors and is either spontaneous or entirely lost, whereas nuclear localization of DROSHA and DGCR8 or cytoplasmic of Ago2 now appears more predominant (Figure 2B,C and Figure S1). Evaluation of these findings across all tumor samples (Figure 2D) confirmed these changes in localization and revealed that: a) they are broad to almost all colon tumors examined; b) they occur in early stages; and c) they persist and become more apparent towards advanced stages (Figure 2D). Importantly, our analysis combined with the analysis of publicly available data from TCGA, shows that E-cadherin is still widely expressed in colorectal tumors, whereas PLEKHA7 is overall downregulated (Figure 2A and Figure S3A,B). Notably, the TCGA data analysis also showed that DROSHA and Ago2 levels are significantly elevated in colon tumors (Figure S3C,D), in addition to the loss of their junctional localization, revealing multiple levels of dysregulation of these proteins correlating with tumor progression. Together, these data demonstrate that a key difference between normal and tumor colon tissues of all stages appears to be the loss of apical junctional or of any cell-cell junction localization of the PLEKHA7-RNAi complex in colon tumors, compared to normal colon epithelial tissues.

### 2.3. PLEKHA7 and RNAi Components Are Mis-Localized in Human Colon Cancer Cell Lines

**Figure 3:**
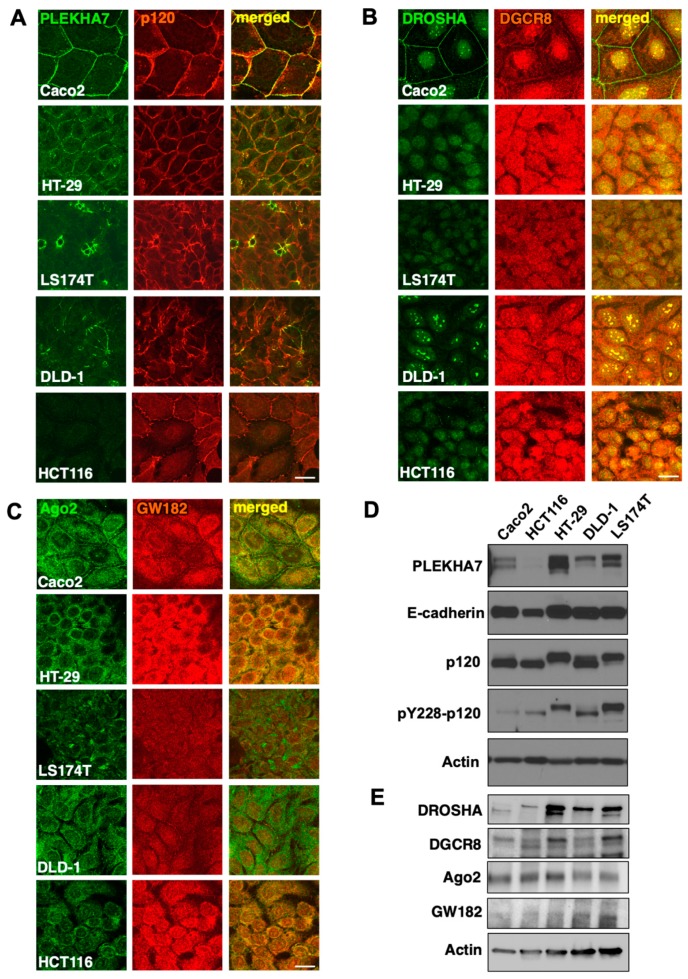
Dysregulation of PLEKHA7 and of RNAi components in colon cancer cells. Well-differentiated colon Caco2 cells and HT-29, DLD-1, LS174T and HCT116 colon cancer cell lines were: (**A**–**C**) stained by immunofluorescence for PLEKHA7, p120, DROSHA, DGCR8, Ago2, GW182 and imaged by confocal microscopy (scale bars: 20 μM); (**D**,**E**) subjected to Western blot for the markers shown. Actin is the loading control.

To follow up on our findings from colon tissues and to obtain further insights into the junctional localization of RNAi components, we examined a panel of colon cancer cells lines, namely DLD-1, HT-29, LS174T, and HCT116. We also included Caco2 cells as a well-differentiated control cell line. More specifically, we performed immunofluorescence and confocal microscopy of these cells for PLEKHA7 and p120 as junctional markers, and for the core RNAi components DROSHA, DGCR8, Ago2, and GW182. Results confirmed that PLEKHA7, as well as all other RNAi proteins that we examined, exhibit junctional localization in Caco2 cells, as we have previously shown (Figure 3A–C). However, junctional localization of PLEKHA7 was compromised in all other cell lines that we examined and appeared fragmented (HT-29), spontaneous (LS174T, DLD-1), or downregulated (HCT116) (Figure 3A–D). Remarkably, and in agreement with the disruption of the junctional localization of PLEKHA7, none of the RNAi components localized at areas of cell-cell contact in any of these cells lines, in stark contrast to the well-differentiated Caco2 cells (Figure 3A–C), but only exhibited predominantly nuclear (DROSHA, DGCR8) or cytoplasmic (Ago2, GW182) localization. This result is in agreement with our data obtained through colon tissue examination (Figure 2) and altogether support the notion that a main distinction between well-differentiated and poorly differentiated colon epithelial cells is the junctional localization of RNAi components.

Importantly, in all these cell lines, p120 and E-cadherin are still expressed and at areas of cell-cell contact (Figure 3A–D), albeit with increased cytoplasmic localization of p120 in HT-29 and HCT116 cells and decreased overall expression of E-cadherin in HCT116 cells. However, in all cases, the loss of junctional localization of RNAi components correlates with the disruption or loss of junctional localization of PLEKHA7, in agreement with our previous published findings showing that PLEKHA7 is required for the recruitment of RNAi components to the adherens junctions [10,25]. Notably, in the three cells lines that PLEKHA7 is still expressed but mis-localized, namely HT-29, DLD-1, LS174T, there is an increase of p120 phosphorylation at Y228, which is phosphorylated by Src (Figure 3D). We have previously shown that Src activity and p120 phosphorylation opposes junctional localization of RNAi components to the junctions [10,25]. Actually, it seems that isoform 1 of p120 is predominant in HT-29 and LS174T cells; this isoform includes multiple tyrosine sites phosphorylated by Src and has been associated with pro-tumorigenic phenotypes [8,32,33,34]. Interestingly, in these three cell lines, HT-29, DLD-1, and LS174T, the overall expression levels of PLEKHA7 seem higher than in Caco2 cells, despite the disruption of its junctional localization, whereas there are also irregularities in the expression pattern of PLEKHA7, with potential differential isoform expression, as revealed by Western blot (Figure 3D).

The loss of junctional localization of RNAi components in the colon cancer cell lines that we examined cannot be attributed to their downregulation, as they are still robustly expressed in these cells, in at least similar levels with Caco2 cells (Figure 3E). In fact, the levels of DROSHA seem to be higher in these cells compared to Caco2 cells (Figure 3E). Interestingly, DROSHA and to some extend DGCR8, seem to also exhibit differential isoform expression. In particular, HT-29, DLD-1, LS174T and HCT116 cells predominantly express a higher molecular weight isoform of DROSHA, compared to Caco2 cells (Figure 3E). These cells also express a lower molecular weight isoform of DGCR8 (Figure 3E). Different isoforms of DROSHA with distinct cytoplasmic or nuclear localization have been recently identified [35,36]. However, it is still not clear whether the isoforms detected here are the same as those previously described. Still, taken together, the data demonstrate that the junctional localization of RNAi components is lost in poorly differentiated colon cancer cells in association with the disruption of junctional localization or loss of expression of PLEKHA7, independently of their overall expression levels or the overall presence of Ecad-p120-mediated cell-cell adhesion. They also reveal several additional factors as candidates for this dysregulation, including different isoforms of these proteins and potential involvement of Src activity.

### 2.4. Src Activity Is Partially Responsible for PLEKHA7-RNAi Mis-Localization in Colon Cancer Cells

**Figure 4:**
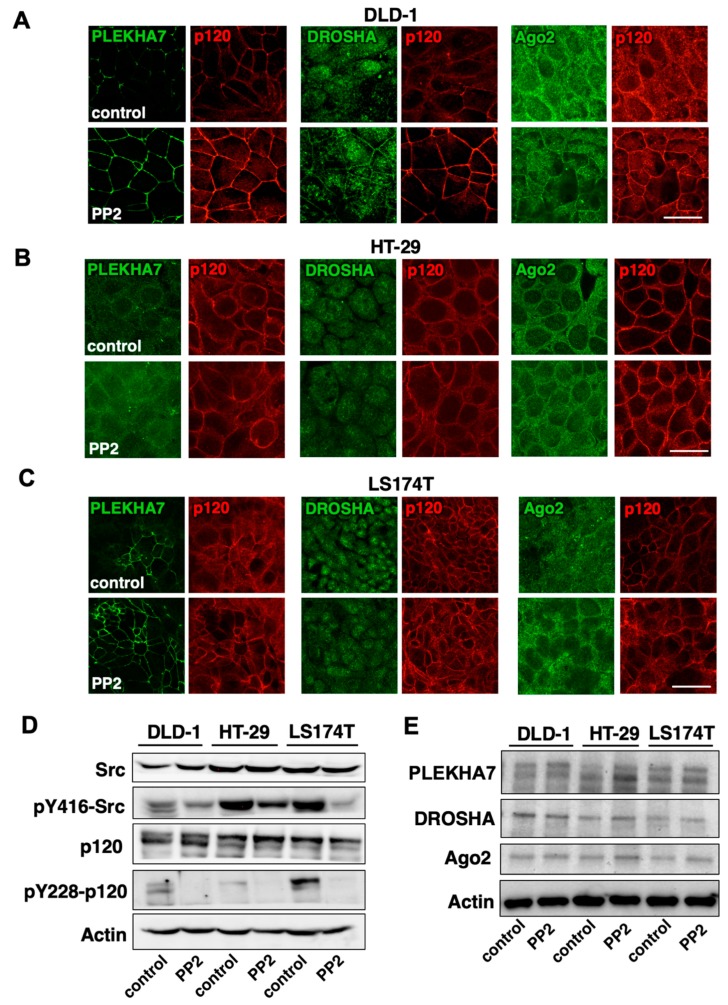
Src activity is partially responsible for the dysregulation of PLEKHA7 and RNAi components in colon cancer cells. HT-29, DLD-1, and LS174T colon cancer cells were treated with the Src inhibitor PP2 or DMSO vehicle (control) for 24 h and: (**A**–**C**) stained by immunofluorescence for PLEKHA7, p120, DROSHA, Ago2 and imaged by confocal microscopy (scale bars: 20 μM); (**D**,**E**) subjected to western blot for the markers shown. Actin is the loading control.

To examine whether Src is indeed responsible for the loss of junctional localization of PLEKHA7 and of RNAi components in HT-29, DLD-1, and LS174T cells, we inhibited Src activity using a chemical inhibitor and examined localization of PLEKHA7, DROSHA, and Ago2, by immunofluorescence and confocal microscopy. Interestingly, Src inhibition restored junctional localization of PLEKHA7, DROSHA, and Ago2 and also strengthened p120 localization to the junctions in DLD-1 cells (Figure 4A). However, it had no effect in any of these markers in HT-29 and LS174T cells (Figure 4B,C), although we confirmed the inhibition of Src phosphorylation at its self-activating Y416 site, as well as the suppression of p120 phosphorylation at the Src-targeting Y228 site in all three cell lines (Figure 4D). Notably, Src inhibition had no effect on overall levels of RNAi proteins examined (Figure 4E). These results demonstrate that Src activity can disrupt junctional localization of PLEKHA7 and of RNAi components, but that there are also additional factors that regulate recruitment of the PLEKHA7-RNAi complex to areas of cell-cell contact, which require further investigation.

### 2.5. PLEKHA7 Re-Expression in HCT116 Cells Restores Junctional Localization of RNAi Components and Suppresses Tumor Growth

**Figure 5:**
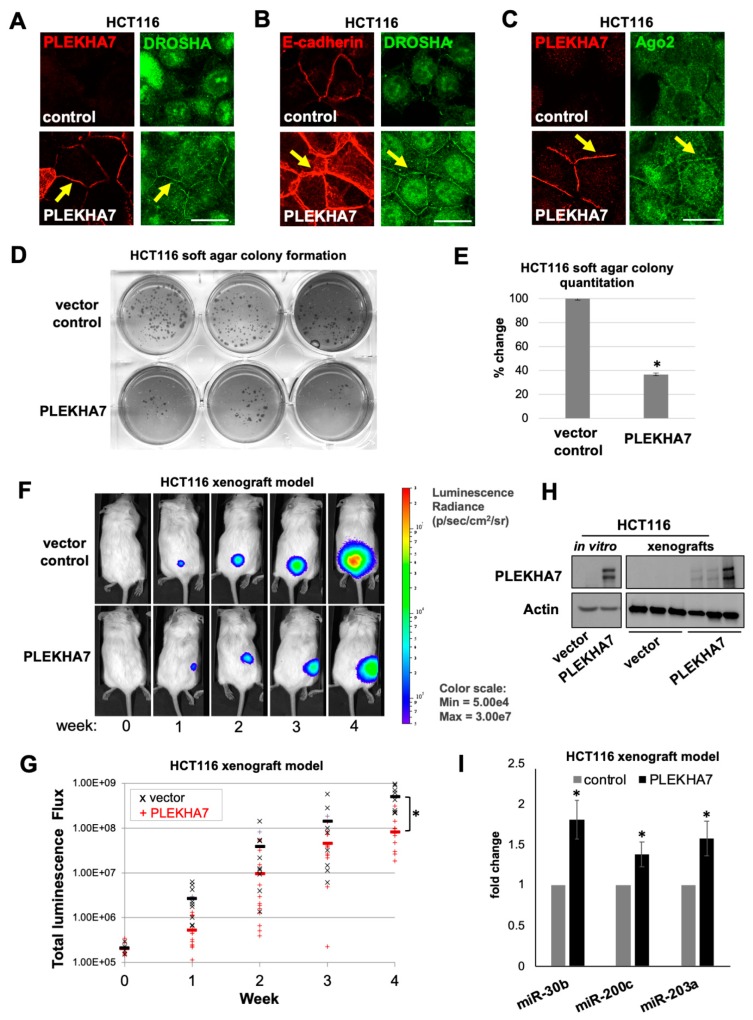
PLEKHA7 re-expression in colon cancer cells restores junctional localization of RNAi and suppresses cancer cell growth in vitro and *in vivo*. HCT116 colon cancer cells that lack endogenous PLEKHA7 expression were virally transduced to express either empty vector control or PLEKHA7. Cells were then: (**A–C**) stained by immunofluorescence for PLEKHA7, E-cadherin, DROSHA, Ago2 and imaged by confocal microscopy (scale bars: 20 μM); (**D**,**E**) subjected to soft agar colony formation assay and colony number quantitation (*n* = 3 independent experiments; *p < 0.05, Student’s two-tailed t-test); (**F**,**G**) engineered to express luciferase and then injected subcutaneously in NOD scid gamma (NSG) mice, were tumor formation was monitored for 4 weeks by using luciferin; image analysis was performed using IVIS, and graphs represent all animals within each group and are analyzed using for total luminescence flux; representative images are shown (*n* = 24 animals, 12 per group, control or PLEKHA7; *p < 0.0012, Student’s two-tailed t-test; both male and female animals were used, *n* = 6 each per experimental group; no significant differences were found between male and female animals); when the 4-week endpoint was reached, mice were euthanized and HCT116 control and PLEKHA7 tumors were extracted and subjected to: (**H**) protein isolation and Western blot analysis, alongside protein lysates of in vitro cultured HCT116 cells, to confirm ectopic expression of PLEKHA7 (Actin is the loading control); and (**I**) RNA isolation and qRT-PCR to examine expression of the miRNAs shown (*n* = 3; *p < 0.02, Student’s two-tailed t-test).

In addition to the three colon cancer cell lines that PLEKHA7 is still expressed in but its junctional localization disrupted, we identified one, namely HCT116, in which PLEKHA7 is downregulated (Figure 3A,D). HCT116 cells are aggressively tumorigenic and highly metastatic [37]; however, they still express E-cadherin and p120 and form cell-cell junctions (Figure 3A,D). We have previously shown that PLEKHA7 depletion promotes pro-tumorigenic transformation of Caco2 cells and results in the disruption of junctional localization of RNAi complexes and downregulation of tumor-suppressing miRNAs, although E-cadherin-mediated cell-cell adhesion is still present [10]. Therefore, we questioned whether the restoration of PLEKHA7 in HCT116 cells would indeed suppress their tumorigenic potential and would be sufficient to restore the junctional localization of RNAi components. Indeed, stable re-expression of PLEKHA7 in HCT116 cells using a retroviral vector strengthens cell-cell adhesion, as visualized by E-cadherin staining, and restores junctional localization of DROSHA and Ago2 (Figure 5A–C). Importantly, PLEKHA7 re-expression significantly suppresses anchorage-independent growth of HCT116 cells in vitro, in a soft agar assay (Figure 5D,E), and tumor growth of these cells in a xenograft mouse model, in vivo (Figure 5F–H). In addition, PLEKHA7 re-expression results in the increased levels of miRNAs that we have previously identified to be regulated by PLEKHA7 in Caco2 cells, such as miR-30b, miR-200c, and miR-203a (Figure 5I) [10,25]. These miRNAs are well-established tumor suppressors and are downregulated in many cancers, including colorectal tumors, as well as in colon cancer cell lines, such as those included in our study [38,39,40,41,42,43,44,45,46,47,48,49,50,51]. Together, these results are in agreement with our previous findings [10,25] and further support a tumor suppressing role for PLEKHA7 in the colonic epithelium, through recruitment and regulation of the RNAi machinery.

## 3. Discussion

Since their discovery, the biochemistry of RNAi complexes has been extensively studied, leading to significant advances towards our understanding of their function [26,27,52,53]. Traditionally, the microprocessor complex, mainly comprised of DROSHA and DGCR8, was considered a solely nuclear component of the cell, whereas the RISC, including Ago2 and GW182, a cytoplasmic one [26,27]. However, our recently published work revealed that the core RNAi complexes are also recruited and localize at the apical adherens junctions of well differentiated epithelial cells, such as human colon Caco2 and canine kidney MDCK cells [10,24,25]. Stemming from the Caco2 findings, the present study sought to investigate the subcellular distribution of the core RNAi components in colon tissues, tumors, and cell lines. Surprisingly, our data not only confirm the apical junctional localization of these complexes but reveal that this localization is actually predominant and a robust feature of normal colon epithelial tissues. These findings signify the physiological relevance of the apical junctional localization of RNAi complexes and further underscore that our findings in Caco2 cells are not a cell line intricacy but rather representative of a widespread characteristic of well-differentiated epithelia.

Strikingly, the junctional localization of RNAi components is entirely lost in colon tumors, even at early stages, or in poorly differentiated colon cancer cell lines. This observation has a number of implications. Firstly, it can explain why the apical junctional localization of RNAi complexes was previously missed; most RNAi-related studies have so far been conducted in transformed cell lines that do not form mature adherens junctions, whereas human tissue studies are scarce and focus on the overall levels of RNAi components, without the systematic examination of their subcellular localization [54]. Secondly, it potentially identifies a novel epithelial-specific biomarker that distinctly separates well-differentiated from transformed cells and tissues, especially since E-cadherin and other adherens junctions markers are still broadly expressed in tumors. Importantly, our findings indicate that the loss of the junctional localization of RNAi is apparent even at early stages, suggesting that it may also be a marker of pro-tumorigenic transformation in polyps or other pre-cancerous conditions, such as inflammatory bowel disease. Thirdly, the loss of junctional RNAi in colon tumors and cell lines, taken together with our previously published findings in Caco2 cells and our current findings on HCT116 cells, suggests that the PLEKHA7-RNAi complex is a potential novel tumor suppressor mechanism, which acts to maintain colonic epithelial homeostasis. Further studies are required to fully understand the tumor-suppressing function of this mechanism and its exact role in colon tumorigenesis.

Cumulatively, the evidence for the junctional association of RNAi complexes in well-differentiated cells and tissues seems now overwhelming, based on our current and previously published findings [10,24,25]. However, we still have little evidence regarding the modes of regulation and the reasons for mis-localization of the junctional RNAi complexes in tumors. The data so far argue that PLEKHA7 is indeed one of the cell-cell adhesion components required for recruitment of these complexes to the junctions [10,25]. PLEKHA7 depletion in Caco2 cells results in the loss of junctional localization of RNAi, whereas PLEKHA7 re-expression in HCT116 cells restores junctional localization of RNAi components [10,25] (Figure 5A). There is also a correlation between the loss of junctional localization of PLEKHA7 and of RNAi in tumor tissues and cell lines (Figure 2 and Figure 3). Still, many questions remain unanswered. It seems that there is both a disruption of junctional localization as well as a downregulation of PLEKHA7 in cells and tissues (Figure 2 and Figure 3). We investigated a potential cause of disruption of junctional localization of PLEKHA7 and RNAi components by examining Src. Src is an oncogene that is extensively involved in colon tumorigenesis [55,56] and is well-established that its activity disrupts strong adhesion [32,57]. Indeed, we have previously shown in Caco2 cells that Src activity opposes junctional localization of RNAi components [10,25]. Our current findings show that Src is only partially responsible for this mis-localization, since its inhibition rescued the junctional localization of the PLEKHA7-RNAi complex only in DLD-1 cells, but not in HT-29 or LS174T cells (Figure 4). Therefore, it seems that other factors regulate the junctional localization of PLEKHA7 and of RNAi components to the junctions that remain to be investigated. In addition, our data show that there is a potentially differential expression of distinct DROSHA and DGCR8 isoforms (Figure 3E). Although the existence of such isoforms for DROSHA and their subcellular localization in the cytoplasm has been recently identified [35,36], their potential to differentially associate with cell-cell adhesion complexes, their exact functions, and their roles during tumorigenesis remain to be clarified. Examination of this and of potential other mechanisms that regulate the junctional localization of RNAi complexes is beyond the scope of the present study, however, it will be the focus of upcoming studies.

The broad dysregulation of the junctional PLEKHA7-RNAi machinery that we have observed, together with the possible existence of multiple modes of regulation and the fact that colon cancer is a multifactorial disease, raise the possibility that the junctional RNAi complex is a focal point at which several pathways converge to sustain colon cell homeostasis and that the dysregulation of either one of them could promote disease progression, through RNAi-miRNA dysregulation. This also suggests that the restoration of this mechanism may also serve as a therapeutic target in the future that could reestablish epithelial homeostasis, which can be disrupted through different oncogenic pathways. Therefore, delineating this network of regulation is essential to be addressed in future studies. In addition, although the present work focuses on colon cells and tissues, it is likely that the association of RNAi with the adherens junctions may be a broad characteristic of epithelial tissues. Indeed, we and others have demonstrated that PLEKHA7 specifically localizes at the apical adherens junctions of other well-differentiated epithelial tissues, such as breast an kidney, and that this localization is broadly disrupted in tumors of these tissues, similarly to our current data on colon tissues [10,20,23]. Therefore, it is also likely that the RNAi components follow the same apical junctional distribution in other well-differentiated epithelia, as in colon tissues. Confirming the prediction that apical junctional localization of RNAi is a universal feature of epithelial tissues with functional consequences will shed new light in our understanding of epithelial homeostasis and will help to better understand numerous diseases, even beyond cancer, and to help design novel therapeutic approaches.

In conclusion, the present study demonstrates that localization of the RNAi machinery at the apical adherens junctions is not only physiologically relevant, but a key property of the differentiated epithelium, at least in the colon. It also reveals that it is broadly dysregulated in tumors and suggests a tumor suppressing role for the PLEKHA7-RNAi complex. Finally, our data provide hints for multiple levels of regulation of this novel mechanism; further investigation is necessary to shed light into these modes of regulation and to clarify the role of the adherens junction-associated RNAi machinery in epithelial homeostasis and function.

## 4. Materials and Methods

### 4.1. Cell Culture, Reagents, and Constructs

All cell lines were acquired from ATCC (Manassas, VA), except human primary colonic epithelial cells (HCnEpC), which were obtained from Cell Applications (Canton, MA, cat# 732Cn-10a). All cells used were tested negative for mycoplasma contamination and they were authenticated by the University of Arizona Genetics Core, via Science Exchange (Palo Alto, CA). In all comparison studies, the cell lines were grown to the same confluences. Cell culture media and supplements (Hyclone), as well as FBS (Life Technologies, Carlsbad, CA) were obtained through Fisher Scientific (Hampton, NH); Caco2 cells were grown in MEM medium containing 10% FBS and 1x each of sodium pyruvate and non-essential amino acids. HCT116 and HT29 cells were grown in McCoy’s 5A containing 10% FBS. DLD-1 cells were grown in RPMI 1640 with 10% FBS. LS174T were grown in MEM medium containing 10% FBS. Phoenix A, a derivative of HEK293 cells used for retroviral production, were grown in DMEM with 10% heat inactivated FBS and 2mM Glutamine. The primary colonic epithelial cells (HCnEpC) do not proliferate in culture and were plated according to the suppliers’ instructions; more specifically, cells were thawed using the GI (gastrointestinal) Epithelial Cell Thawing Solution (Cell Applications, Canton, MA, cat #716T-20), centrifuged at 200g for 5 min and resuspended in the GI Epithelial Cell Defined Culture Medium (Cell Applications, Canton, MA, cat #716DC-50), and then plated on coverslips that were pre-coated using the GI Epithelial Cell Coating Solution Cell Applications, Canton, MA, cat #025-05). In order for cells to be confluent, 5 × 10^5^ cells/coverslip were plated on 12 mm round coverslips and allowed to form cell-cell contacts for 48 hr before fixation and immunofluorescence.

Retroviral control LZRS and PLEKHA7 expressing (LZRS-PLEKHA7) vectors were used before [10]. Viral particles were generated in Phoenix A packaging cells and used to infect HCT116 cells. Cells with stable retroviral integration were selected using G418 for 7–10 days. The detailed protocol was described previously [10]. For mouse xenograft experiments, HCT116-LZRS and HCT116-LZRS-PLEKHA7 cells were also infected with lentiviral vector pLX304 Luciferase-V5Blast (Addgene, Watertown, MA, cat# 98580) [58] for constitutive expression of luciferase, to allow in vivo detection in the mice. These will be referred to hence forth as HCT116-LZRS-LUC and HCT116LZRS-PLEKHA7-LUC. The Src inhibitor PP2 was obtained from Sigma, St Louis, MO (cat #529573).

### 4.2. Antibodies

Primary antibodies used in the present study are: PLEKHA7 (Sigma, St Louis, MO, cat# HPA038610), p120 (EMD Millipore, Burlington, MA, cat# 05-1567), E-cadherin (Life Technologies, Carlsbad, CA, cat# 18-0223), Ago2 (Abcam, Cambridge, MA, cat# ab57113; Novus Biologicals, Centennial CO, cat# 28550002; ECM Biosciences, Versailles, KY, cat# AP5281), GW182 (Santa Cruz, Dallas, TX, cat# sc-56314), DROSHA (Life Technologies, Carlsbad, CA, cat# PA25-97013; Life Technologies, Carlsbad, CA, cat# MA5-3281; Abcam, Cambridge, MA, cat# Ab12216; Abnova, Taipei, Taiwan, cat# PAB7156), DGCR8 (Sigma, St Louis, MO, cat# HPA019965; Abnova, Taipei, Taiwan, cat# H00054487-M01), Actin (Sigma, St Louis, MO, cat# A2066). Working dilutions: 1:50-1:500 for immunofluorescence, 1:500-1:2000 for Western blot. Secondary antibodies used in the present study: HRP-anti-mouse (Jackson ImmunoResearch, West Grove, PA, cat# 715-035-150), HRP-anti-rabbit (Jackson ImmunoResearch, West Grove, PA, cat# 711-035-152), Alexa 488 anti-mouse (Life Technologies, Carlsbad, CA, cat# A-11029), Alexa 488 anti-rabbit (Life Technologies, Carlsbad, CA, cat# A11034), Alexa 594 anti-mouse (Life Technologies, Carlsbad, CA, cat# A-11005), Alexa 594 anti-rabbit (Life Technologies, Carlsbad, CA, cat# A-11037). Working dilutions: 1:500 for immunofluorescence, 1:2000 for Western blot.

### 4.3. Western Blot

Whole cell protein extracts were obtained using RIPA buffer (Tris, pH 7.4, 50 mM NaCl, 150 mM, NP-40, 1%, Deoxycholic Acid, 0.5%, SDS, 0.1%) supplemented with protease (cocktail III; RPI, Mount Prospect, IL) and phosphatase inhibitors (Pierce, Hampton, NH). Lysates were homogenized through a 29 g needle and cleared by full speed centrifugation for 5 min. Protein quantification was performed using the BCA assay (Pierce, Hampton, NH). Protein extracts were mixed with Laemmli Sample Buffer (LSB) and separated using Mini-PROTEAN TGX (4–15%) gels (Bio-Rad, Hercules, CA) transferred using Trans-Blot Turbo Transfer (Bio-Rad, Hercules, CA), blotted according to standard protocols, detected by luminescence using ECL (GE Healthcare Hampton, NH) and imaged using a Bio-Rad ChemiDoc (Hercules, CA). For xenograft tumor protein extraction, tumors were crushed using a dry-ice, pre-chilled pestle and mortar, followed by 3 × 10 sec sonication in chilled conditions, before protein extraction was performed as above.

### 4.4. Cultured Cells Immunofluorescence

Cells were grown on sterile glass coverslips until they were at least 90% confluent. Cells were washed once with PBS and then fixed with either: i) 100% methanol (Fisher Scientific, Hampton, NH) for 7 min at −20 °C; or ii) 4% formaldehyde (Fisher Scientific, Hampton, NH, cat # 50-259-99) for 20 min at RT, followed by 0.02% Triton-X 100 permeabilization for 10 min. Cells were blocked with Protein-Block reagent (Dako, Santa Clara, CA, cat # X090930-2) for 60 min and stained with primary antibodies diluted in Antibody Diluent (Dako, Santa Clara, CA, cat # S302281-2) for 1 h. Cells were then washed three times with PBS, stained with the fluorescent-labelled secondary antibodies, protected from light, for 1 h, washed three times with PBS, co-stained with DAPI (Sigma, St Louis, MO) to visualize the nuclei, mounted (Aqua Poly/Mount, Polysciences, Warrington, PA, cat # 18606), and imaged using a Leica SP5 confocal microscope, under a 63× objective, with an additional 1.5× zoom. Z-stacks were acquired in 0.5–1.0 μM intervals. Images and stacks were analyzed using the ImageJ software.

### 4.5. Human Tissue Collection and Immunofluorescence—Immunohistochemistry

Colon tissue samples were obtained from the Hollings Cancer Center Biorepository, Medical University of South Carolina (MUSC), under protocol number Pro00062968 (02-02-2017), with the approval of the MUSC’s Institutional Review Board. Tissue samples were de-identified (unique patient identifiers and confidential data removed) before samples were obtained; thus the study was deemed as Not Human Research (NHR). Fresh frozen tissues were air-dried at room temperature for 10 min and fixed in acetone (Fischer Chemical, Hampton, NH, cat # A18-1) for 20 min at −20 °C. Then, samples were rehydrated in PBS (Corning, Hampton, NH, cat # 21-040-CV) three times. A hydrophobic barrier was marked around tissues using Aqua Hold II pen (Scientific Device Laboratory, Des Plaines, IL, cat # 9804-02). Afterwards, samples were blocked with a blocking reagent (Dako, Santa Clara, CA, cat # X090930-2) for 1 h at room temperature and stained with primary antibodies diluted in an Antibody diluent (Dako, Santa Clara, CA, cat # S302281-2) for 2 h at room temperature. Samples were then washed three times with PBS and stained with fluorescent-labeled secondary antibodies diluted in an Antibody diluent (Dako, Santa Clara, CA, cat # S302281-2) for 1 h at room temperature. Samples were then washed two times with PBS, co-stained with DAPI (Sigma, St Louis, MO, cat # D8417) diluted in PBS and washed once again with PBS. Slides were then mounted (Aqua Poly/Mount, Polysciences, Warrington, PA, cat # 18606) and were dried overnight at room temperature in the dark.

Tissues slides were also subjected to H&E stainings for histology evaluation of tumor areas. For this, slides were immersed in xylene (Fisher Scientific, Hampton, NH) twice for 5 min each time and were rehydrated through a series of EtOH (100-100-95-80-70-50%) and placed in water. Samples were then stained with hematoxylin (Polysciences, Warrington, PA, cat # 24243-500) for 2 min and washed in water-acid alcohol-water. Samples were then immersed in blue ammonia water for 1 min, washed with water, placed in 95% EtOH for 1 min and stained with in eosin Y solution (Fisher Scientific, Hampton, NH, cat # 45380-M1159350100) for 1 min. Samples were then rapidly dehydrated through a series of EtOH (95-95-100-100%), placed in xylenes twice for 5 min each time and were mounted with Cytoseal (Fisher Scientific, Hampton, NH, cat # 831016).

Commercially available paraffin-embedded colon tissue microarrays (TMAs) were obtained from US Biomax (Rockville, MD, cat# OD-CT-DgCol04-003). Slides were deparaffinized by immersing in xylene (Fisher Scientific, Hampton, NH) twice for 5 min each time. Samples were then rehydrated through a series of EtOH (100-100-95-80-70-50%) and placed in distilled water. Samples were then subjected to an antigen retrieval using a mix (Vector Laboratories, Burlingame, CA, cat # H-3300) for 5 min, and were rinsed in distilled water and in PBS (Corning, Hampton, NH, cat # 21-040-CV) respectively. Afterwards, samples were blocked with a blocking reagent (Dako, Santa Clara, CA, cat # X090930-2;) for 1 h at room temperature in a humid chamber and stained with primary antibodies diluted in an Antibody diluent (Dako, Santa Clara, CA, cat # S302281-2) overnight at 4 °C in a humid chamber. Samples were then washed three times with PBS and stained with fluorescent-labeled secondary antibodies diluted in an Antibody diluent (Dako, Santa Clara, CA, cat # S302281-2) for 1 h at room temperature in a humid chamber. Next, samples were washed three times with PBS and mounted using Slowfade with DAPI (Fisher Scientific Hampton, NH, cat # S36964).

Primary antibodies specifically used for tissue immunostainings were: PLEKHA7 (Sigma, St Louis, MO, cat # HPA038610), DROSHA (Abnova, Taipei, Taiwan, cat # PAB7156), DGCR8 (Abnova, Taipei, Taiwan, cat # H00054487-M01), Ago2 (Santa Cruz, Dallas, TX, cat # sc376696) and E-cadherin (4A2C7; Life Technologies, Carlsbad, CA, cat # 180223). Working dilutions were 1:50 to 1:500. Secondary antibodies used in the study were Alexa 488 anti-mouse (Life Technologies, Carlsbad, CA, cat # A21202), Alexa 488 anti-rabbit (Life Technologies, Carlsbad, CA, cat # A21206), Alexa 594 anti-rabbit (Life Technologies, Carlsbad, CA, cat # A11037) and Alexa 594 anti-goat (Life Technologies, Carlsbad, CA, cat # A11058). The working dilution was 1:500. Slides were imaged using a Vectra Multispectral Imaging System (Perkin-Elmer, Waltham, MA) under a 20x objective and analyzed using QuPath v0.1.2 [59]; or imaged using a Leica SP5 confocal microscope, under a 63x objective, with an additional 1.5x zoom. The analysis was qualitative, evaluating apical or junctional localization of the markers under examination.

### 4.6. Mouse Xenograft Experiments

All animal experiments were performed according to policies and guidelines of the Institutional Animal Care and Use Committee (IACUC) at MUSC under the approved protocol IACUC-2018-00438 (04-23-2019). Six- to eight-week-old male and female NOD scid gamma (NSG) mice (Jackson labs, Bar Harbor, ME) were used. All working surfaces were disinfected by wiping with 70% ethanol. The anesthesia chamber was set up with oxygen/isoflurane mixture according to manufacturer’s instructions: 2.5% (*v/v*) isoflurane to achieve depth of anesthesia and 1% (*v/v*) isoflurane to maintain anesthetic plane. The isoflurane (Fisher Scientific Hampton, NH) was obtained through the MUSC Pharmacy. Fifty thousand (50,000) cells in 50 ul of HBSS 1× (Gibco, cat # 14170-120; Fisher Scientific, Hampton, NH) of either HCT116 LZRS-LUC (control) or HCT116-LZRS-PLEKHA7-LUC cells were injected subcutaneously into either the left or right flank of each mouse. Tumor burden and location were evaluated using luciferase activity. For bioluminescence imaging, mice were anesthetized as above: 2.5% (*v/v*) isoflurane to depth of anesthesia and maintained with 1% (*v/v*) isoflurane during imaging protocols. D-Luciferin (Fisher Scientific, Hampton NH, cat # NC 1276267; 150 μg mL-1; substrate for luciferase) was injected within the peritoneal cavity. Bioluminescence images (BLI) and measurements were taken using an IVIS 200 imager (PerkinElmer, Waltham, MA). BLI were taken one day post-tumor implantation and then weekly and up to four weeks post-implantation. Acquisitions were taken 1, 5, 10, 15, and 20 min after luciferin injection. To standardize the data, a region of interest (ROI) was drawn around each tumor at each longitudinal imaging timepoint. Light emission was quantified from the same surface area (ROI) for each tumor. Corresponding gray scale photographs and color luminescence images were superimposed and luminescence analyzed using Living Image analysis software version 3.1 (Caliper Life Sciences, Waltham, MA). The data were calculated using Average Radiance, which is the sum of the radiance from each pixel inside the region of interest/number of pixels (photons/sec/cm^2/sr) and scaled the same for all data longitudinal time points. Representative images are shown; graphs represent all animals within each group and are statistically analyzed using standard analysis of variance techniques. The experimental end point was determined through preliminary experiments using control HCT116 cells at 4 weeks, which is right before the tumor size renders animals morbid. At the end point, animals were sacrificed using CO2 and cervical dislocation and tumors were excised for further analysis.

### 4.7. RNA Isolation and qRT-PCR

Cells from cultured plates or tumors from xenografts were lysed using Trizol (Invitrogen, Carlsbad, CA) and subjected to the Trizol Plus Total Transcriptome Isolation protocol of the PureLink RNA mini kit (Ambion - Life Technologies, Carlsbad, CA). Final RNA concentrations were determined using a BioDrop uLite spectrophotometer (Harvard BioScience, Holliston, MA). RNA was converted to cDNA using the High Capacity cDNA Reverse Transcriptase Kit (Applied Biosystems, Waltham, MA). qPCR reactions were performed using the Taqman FAST Universal PCR master mix (Applied Biosystems, Waltham, MA) in a Bio-Rad CFX96 qPCR thermocycler (Hercules, CA). U6 was used as a control for miRNA expression normalization. TaqMan assays used for miRNAs (Applied Biosystems, Waltham, MA, cat# 4427975): hsa-miR-30b, 000602; hsa-miR-200c, 002300; hsa-miR-203a, 000507; U6, 001973.

### 4.8. Soft Agar Assay

Six 6-well plates (Costar, 3516, Corning, NY) were layered with 0.75% agarose, prepared by mixing 1.5% of sterile agarose (Fisher Scientific, Hampton NH, cat # BP1356100) in a 1:1 ratio with 2× concentrated HCT116 culture medium made from powder McCoy’s (Sigma, St Louis, MO, cat # M4892) and double addition of each supplement (see above). The basal layer was left to solidify for 30 min. Cells were trypsinized, counted, and re-suspended in 2x medium. The cell suspension was mixed in a 1:1 ratio with 0.7% sterile agarose to make a final layer of 0.35% agarose, which was added on top of the basal layer. 5 × 10^4^ HCT116 cells were seeded per well. The top layer was left to solidify for another 30 min and was covered with 1× culture medium. Cultures were maintained for 2 to 3 weeks with medium renewal on top of agar every three days, until colonies were visible and then stained with 0.02% Crystal violet (Fisher Scientific, Hampton NH) in 20% Ethanol and PBS, washed three times with distilled H_2_O, scanned, and counted for colonies.

### 4.9. TCGA Data Analysis

Data extraction and analysis from the Cancer Genome Atlas (TCGA) was performed using the online Gene Expression Profiling Interactive Analysis (GEPIA) GEPIA [60]: http://gepia.cancer-pku.cn. We used the Box Plot drawing option of the Expression DIY module of GEPIA. To analyze data, we selected the colon (COAD) and rectal (READ) datasets and used a Log2FC Cutoff value of 1 and a *p*-value cutoff of 0.01. The total number of tissues analyzed were: colon tumors, 275; colon normal, 41; rectal tumors, 92; rectal normal, 10.

### 4.10. Statistics and Reproducibility

For all quantitative experiments, averages and standard deviations (s.d.) were calculated and presented as error bars; the number of independent experiments performed, sample size, and the related statistics are indicated in each figure legend. The Student’s two-tailed t-test was employed for *p* value calculations since all comparisons were between two groups, control and experimental condition. For all other experiments, at least three independent experiments were performed; a representative is shown in each Figure.

## Supplementary Materials

The following are available online at https://www.mdpi.com/1422-0067/21/7/2559/s1.

## Abbreviations

Ago2	Argonaute RISC Catalytic Component 2
BLI	Bioluminescence imaging
DAPI	4′,6-diamidino-2-phenylindole
DGCR8	DiGeorge Syndrome Critical Region Gene 8
DROSHA	Drosha Ribonuclease III
ECL	Enhanced Chemiluminescence
GW182	Glycine-Tryptophan Protein Of 182 KDa
HBSS	Hank’s Balanced Salt Solution
HEK293	Human Embryonic Kidney 293 cells
HRP	Horseradish Peroxidase
IACUC	Institutional Animal Care and Use Committee
LSB	Laemmli Sample Buffer
MDCK	Madin-Darby Canine Kidney cells
miRNA	microRNA
MUSC	Medical University of South Carolina
NSG	NOD scid gamma
p120	p120 catenin
PBS	Phosphate-Buffered Saline
PLEKHA7	Pleckstrin Homology Domain Containing A7
qRT-PCR	quantitative reverse transcription polymerase chain reaction
RIPA	Radioimmunoprecipitation assay buffer
RISC	RNA-induced silencing complex
RNA	Ribonucleic Acid
RNAi	RNA interference
TCGA	The Cancer Genome Atlas

## References

[1] Siegel R.L., Miller K.D., Jemal A. (2019). Cancer statistics, 2019. CA: A Cancer J. Clin..

[2] Chakradhar S. (2015). Colorectal cancer: 5 big questions. Nature.

[3] Coskun M. (2014). Intestinal epithelium in inflammatory bowel disease. Front. Med..

[4] Landy J., Ronde E., English N., Clark S.K., Hart A.L., Knight S.C., Ciclitira P.J., Al-Hassi H.O. (2016). Tight junctions in inflammatory bowel diseases and inflammatory bowel disease associated colorectal cancer. World J. Gastroenterol..

[5] Daulagala A.C., Bridges M.C., Kourtidis A. (2019). E-cadherin Beyond Structure: A Signaling Hub in Colon Homeostasis and Disease. Int. J. Mol. Sci..

[6] Takeichi M. (2014). Dynamic contacts: Rearranging adherens junctions to drive epithelial remodelling. Nat. Rev. Mol. Cell Biol..

[7] Harris T.J., Tepass U. (2010). Adherens junctions: From molecules to morphogenesis. Nat. Rev. Mol. Cell Biol..

[8] Kourtidis A., Ngok S.P., Anastasiadis P.Z. (2013). p120 catenin: An essential regulator of cadherin stability, adhesion-induced signaling, and cancer progression. Prog. Mol. Biol. Transl. Sci..

[9] Kourtidis A., Lu R., Pence L.J., Anastasiadis P.Z. (2017). A central role for cadherin signaling in cancer. Exp. Cell Res..

[10] Kourtidis A., Ngok S.P., Pulimeno P., Feathers R.W., Carpio L.R., Baker T.R., Carr J.M., Yan I.K., Borges S., Perez E.A. (2015). Distinct E-cadherin-based complexes regulate cell behaviour through miRNA processing or Src and p120 catenin activity. Nat. Cell Biol..

[11] Dohn M.R., Brown M.V., Reynolds A.B. (2009). An essential role for p120-catenin in Src- and Rac1-mediated anchorage-independent cell growth. J. Cell Biol..

[12] Shamir E.R., Pappalardo E., Jorgens D.M., Coutinho K., Tsai W.T., Aziz K., Auer M., Tran P.T., Bader J.S., Ewald A.J. (2014). Twist1-induced dissemination preserves epithelial identity and requires E-cadherin. J. Cell Biol..

[13] Padmanaban V., Krol I., Suhail Y., Szczerba B.M., Aceto N., Bader J.S., Ewald A.J. (2019). E-cadherin is required for metastasis in multiple models of breast cancer. Nature.

[14] Gritsenko P.G., Atlasy N., Dieteren C.E.J., Navis A.C., Venhuizen J.H., Veelken C., Schubert D., Acker-Palmer A., Westerman B.A., Wurdinger T. (2020). p120-catenin-dependent collective brain infiltration by glioma cell networks. Nat. Cell Biol..

[15] Lewis-Tuffin L.J., Rodriguez F., Giannini C., Scheithauer B., Necela B.M., Sarkaria J.N., Anastasiadis P.Z. (2010). Misregulated E-cadherin expression associated with an aggressive brain tumor phenotype. PLoS ONE.

[16] Rodriguez F.J., Lewis-Tuffin L.J., Anastasiadis P.Z. (2012). E-cadherin’s dark side: Possible role in tumor progression. Biochim. Biophys. Acta.

[17] Kuphal S., Bosserhoff A.K. (2012). E-cadherin cell-cell communication in melanogenesis and during development of malignant melanoma. Arch. Biochem. Biophys..

[18] Liu W.F., Nelson C.M., Pirone D.M., Chen C.S. (2006). E-cadherin engagement stimulates proliferation via Rac1. J. Cell Biol..

[19] Meng W., Mushika Y., Ichii T., Takeichi M. (2008). Anchorage of microtubule minus ends to adherens junctions regulates epithelial cell-cell contacts. Cell.

[20] Pulimeno P., Bauer C., Stutz J., Citi S. (2010). PLEKHA7 is an adherens junction protein with a tissue distribution and subcellular localization distinct from ZO-1 and E-cadherin. PLoS ONE.

[21] Kourtidis A., Anastasiadis P.Z. (2016). PLEKHA7 defines an apical junctional complex with cytoskeletal associations and miRNA-mediated growth implications. Cell Cycle.

[22] Rea K., Roggiani F., De Cecco L., Raspagliesi F., Carcangiu M.L., Nair-Menon J., Bagnoli M., Bortolomai I., Mezzanzanica D., Canevari S. (2018). Simultaneous E-cadherin and PLEKHA7 expression negatively affects E-cadherin/EGFR mediated ovarian cancer cell growth. J. Exp. Clin. Cancer Res..

[23] Tille J.C., Ho L., Shah J., Seyde O., McKee T.A., Citi S. (2015). The Expression of the Zonula Adhaerens Protein PLEKHA7 Is Strongly Decreased in High Grade Ductal and Lobular Breast Carcinomas. PLoS ONE.

[24] Kourtidis A., Anastasiadis P.Z. (2018). Close encounters of the RNAi kind: The silencing life of the adherens junctions. Curr. Opin. Cell Biol..

[25] Kourtidis A., Necela B., Lin W.H., Lu R., Feathers R.W., Asmann Y.W., Thompson E.A., Anastasiadis P.Z. (2017). Cadherin complexes recruit mRNAs and RISC to regulate epithelial cell signaling. J. Cell Biol..

[26] Ha M., Kim V.N. (2014). Regulation of microRNA biogenesis. Nat. Rev. Mol. Cell Biol..

[27] Krol J., Loedige I., Filipowicz W. (2010). The widespread regulation of microRNA biogenesis, function and decay. Nat. Reviews. Genet..

[28] Grasset E., Pinto M., Dussaulx E., Zweibaum A., Desjeux J.F. (1984). Epithelial properties of human colonic carcinoma cell line Caco-2: Electrical parameters. Am. J. Physiol.

[29] Hidalgo I.J., Raub T.J., Borchardt R.T. (1989). Characterization of the human colon carcinoma cell line (Caco-2) as a model system for intestinal epithelial permeability. Gastroenterology.

[30] Sambuy Y., De Angelis I., Ranaldi G., Scarino M.L., Stammati A., Zucco F. (2005). The Caco-2 cell line as a model of the intestinal barrier: Influence of cell and culture-related factors on Caco-2 cell functional characteristics. Cell Biol. Toxicol..

[31] Humphries A., Wright N.A. (2008). Colonic crypt organization and tumorigenesis. Nat. Rev..

[32] Ozawa M., Ohkubo T. (2001). Tyrosine phosphorylation of p120(ctn) in v-Src transfected L cells depends on its association with E-cadherin and reduces adhesion activity. J. Cell Sci..

[33] Ohkubo T., Ozawa M. (2004). The transcription factor Snail downregulates the tight junction components independently of E-cadherin downregulation. J. Cell Sci..

[34] Shapiro I.M., Cheng A.W., Flytzanis N.C., Balsamo M., Condeelis J.S., Oktay M.H., Burge C.B., Gertler F.B. (2011). An EMT-driven alternative splicing program occurs in human breast cancer and modulates cellular phenotype. PLoS Genet..

[35] Link S., Grund S.E., Diederichs S. (2016). Alternative splicing affects the subcellular localization of Drosha. Nucleic Acids Res..

[36] Dai L., Chen K., Youngren B., Kulina J., Yang A., Guo Z., Li J., Yu P., Gu S. (2016). Cytoplasmic Drosha activity generated by alternative splicing. Nucleic Acids Res..

[37] Rajput A., Dominguez San Martin I., Rose R., Beko A., Levea C., Sharratt E., Mazurchuk R., Hoffman R.M., Brattain M.G., Wang J. (2008). Characterization of HCT116 human colon cancer cells in an orthotopic model. J. Surg. Res..

[38] Joglekar M.V., Patil D., Joglekar V.M., Rao G.V., Reddy D.N., Mitnala S., Shouche Y., Hardikar A.A. (2009). The miR-30 family microRNAs confer epithelial phenotype to human pancreatic cells. Islets.

[39] Zhang J., Zhang H., Liu J., Tu X., Zang Y., Zhu J., Chen J., Dong L., Zhang J. (2012). miR-30 inhibits TGF-beta1-induced epithelial-to-mesenchymal transition in hepatocyte by targeting Snail1. Biochem. Biophys. Res. Commun..

[40] Mutlu M., Raza U., Saatci O., Eyupoglu E., Yurdusev E., Sahin O. (2016). miR-200c: A versatile watchdog in cancer progression, EMT, and drug resistance. J. Mol. Med. (Berl).

[41] Lohcharoenkal W., Das Mahapatra K., Pasquali L., Crudden C., Kular L., Akkaya Ulum Y.Z., Zhang L., Xu Landen N., Girnita L., Jagodic M. (2018). Genome-Wide Screen for MicroRNAs Reveals a Role for miR-203 in Melanoma Metastasis. J. Investig. Dermatol..

[42] Liu K., Chen W., Lei S., Xiong L., Zhao H., Liang D., Lei Z., Zhou N., Yao H., Liang Y. (2017). Wild-type and mutant p53 differentially modulate miR-124/iASPP feedback following pohotodynamic therapy in human colon cancer cell line. Cell Death Dis..

[43] Qian Z., Gong L., Mou Y., Han Y., Zheng S. (2019). MicroRNA203a3p is a candidate tumor suppressor that targets thrombospondin 2 in colorectal carcinoma. Oncol. Rep..

[44] Ogawa H., Wu X., Kawamoto K., Nishida N., Konno M., Koseki J., Matsui H., Noguchi K., Gotoh N., Yamamoto T. (2015). MicroRNAs Induce Epigenetic Reprogramming and Suppress Malignant Phenotypes of Human Colon Cancer Cells. PLoS ONE.

[45] Liao W.T., Ye Y.P., Zhang N.J., Li T.T., Wang S.Y., Cui Y.M., Qi L., Wu P., Jiao H.L., Xie Y.J. (2014). MicroRNA-30b functions as a tumour suppressor in human colorectal cancer by targeting KRAS, PIK3CD and BCL2. J. Pathol..

[46] Zhao H., Xu Z., Qin H., Gao Z., Gao L. (2014). miR-30b regulates migration and invasion of human colorectal cancer via SIX1. Biochem. J..

[47] Hur K., Toiyama Y., Takahashi M., Balaguer F., Nagasaka T., Koike J., Hemmi H., Koi M., Boland C.R., Goel A. (2013). MicroRNA-200c modulates epithelial-to-mesenchymal transition (EMT) in human colorectal cancer metastasis. Gut.

[48] Karimi Dermani F., Amini R., Saidijam M., Najafi R. (2018). miR-200c, a tumor suppressor that modulate the expression of cancer stem cells markers and epithelial-mesenchymal transition in colorectal cancer. J. Cell. Biochem..

[49] Karimi Dermani F., Najafi R. (2018). miR-200c as a Predictive Biomarker for 5-Fluorouracil Chemosensitivity in Colorectal Cancer. J. Gastrointest Cancer.

[50] Wang Z.Z., Yang J., Jiang B.H., Di J.B., Gao P., Peng L., Su X.Q. (2018). KIF14 promotes cell proliferation via activation of Akt and is directly targeted by miR-200c in colorectal cancer. Int. J. Oncol..

[51] Lu Y.X., Yuan L., Xue X.L., Zhou M., Liu Y., Zhang C., Li J.P., Zheng L., Hong M., Li X.N. (2014). Regulation of colorectal carcinoma stemness, growth, and metastasis by an miR-200c-Sox2-negative feedback loop mechanism. Clin. Cancer Res..

[52] Gebert L.F.R., MacRae I.J. (2019). Regulation of microRNA function in animals. Nat. Rev. Mol. Cell Biol..

[53] Meister G. (2013). Argonaute proteins: Functional insights and emerging roles. Nat. Rev. Genet..

[54] Vychytilova-Faltejskova P., Svobodova Kovarikova A., Grolich T., Prochazka V., Slaba K., Machackova T., Halamkova J., Svoboda M., Kala Z., Kiss I. (2019). MicroRNA Biogenesis Pathway Genes Are Deregulated in Colorectal Cancer. Int. J. Mol. Sci..

[55] Chen J., Elfiky A., Han M., Chen C., Saif M.W. (2014). The role of Src in colon cancer and its therapeutic implications. Clin. Colorectal Cancer.

[56] Chen J. (2008). Is Src the key to understanding metastasis and developing new treatments for colon cancer?. Nat. Clin. Pr. Gastroenterol. Hepatol..

[57] Mariner D.J., Anastasiadis P., Keilhack H., Bohmer F.D., Wang J., Reynolds A.B. (2001). Identification of Src phosphorylation sites in the catenin p120ctn. J. Biol. Chem..

[58] Bajikar S.S., Wang C.C., Borten M.A., Pereira E.J., Atkins K.A., Janes K.A. (2017). Tumor-Suppressor Inactivation of GDF11 Occurs by Precursor Sequestration in Triple-Negative Breast Cancer. Dev. Cell.

[59] Bankhead P., Loughrey M.B., Fernandez J.A., Dombrowski Y., McArt D.G., Dunne P.D., McQuaid S., Gray R.T., Murray L.J., Coleman H.G. (2017). QuPath: Open source software for digital pathology image analysis. Sci Rep..

[60] Tang Z., Li C., Kang B., Gao G., Li C., Zhang Z. (2017). GEPIA: A web server for cancer and normal gene expression profiling and interactive analyses. Nucleic Acids Res..

