# Supplementary material for: Predominant Distribution of the RNAi Machinery at Apical Adherens Junctions in Colonic Epithelia Is Disrupted in Cancer
Source: Int J Mol Sci. 2020 Apr 7;21(7):2559. doi: 10.3390/ijms21072559 (PMC7177752; doi:10.3390/ijms21072559)
Supplement: Supplementary file 1 [file ijms-21-02559-s001.pdf]

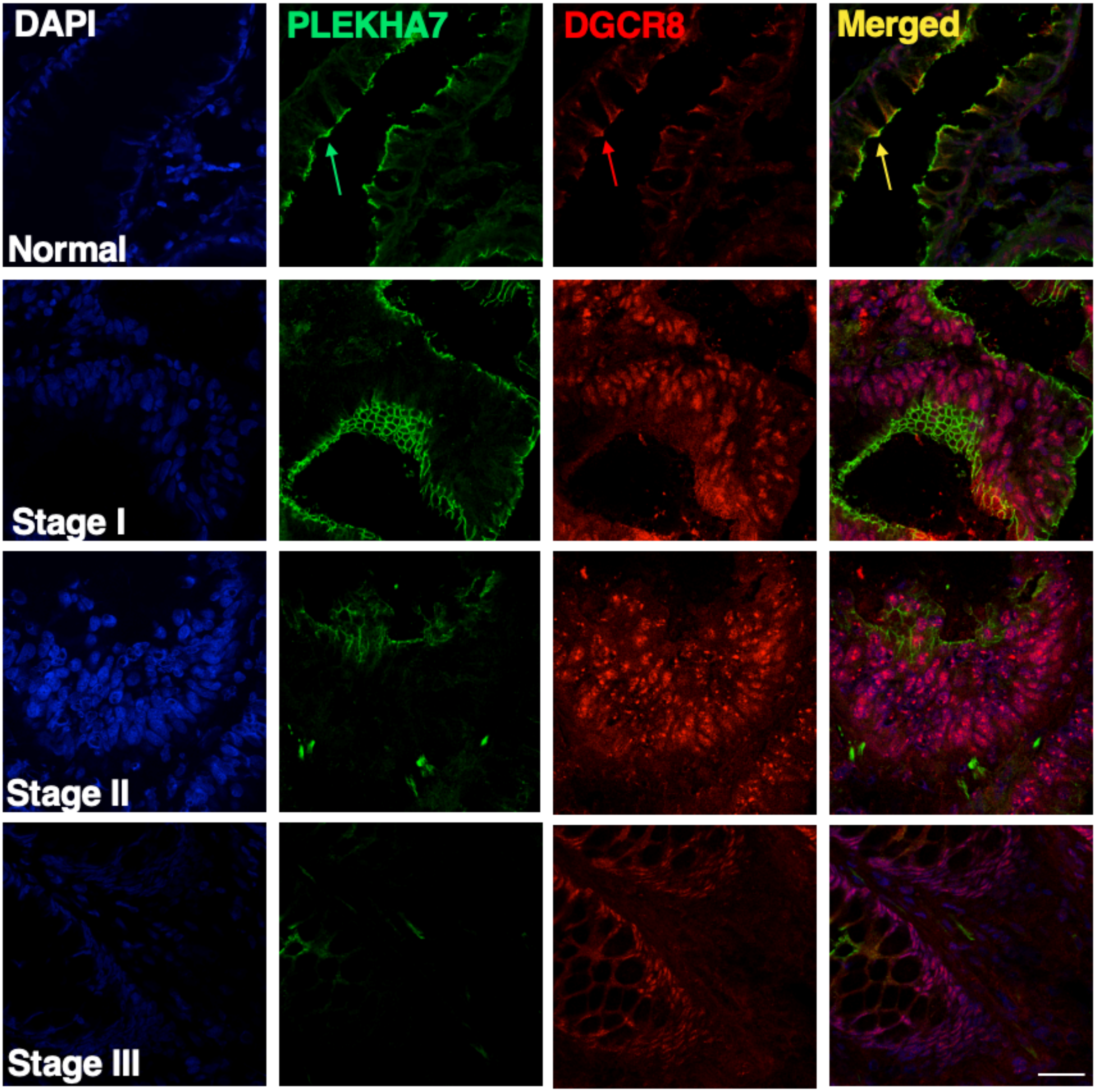


**Figure S1. The RNAi machinery localizes predominantly at apical junctions in normal colon tissues but is dysregulated in colon cancer.** Examination of a total of colon normal and matched tumor tissues from 33 patient tissue samples to assess localization status of RNAi machinery components. Immunofluorescence staining for PLEKHA7 and DGCR8. DAPI is the nuclear stain. Representative tissues are shown. Arrows indicate apical localization. Scale bar: 20 μM.

A

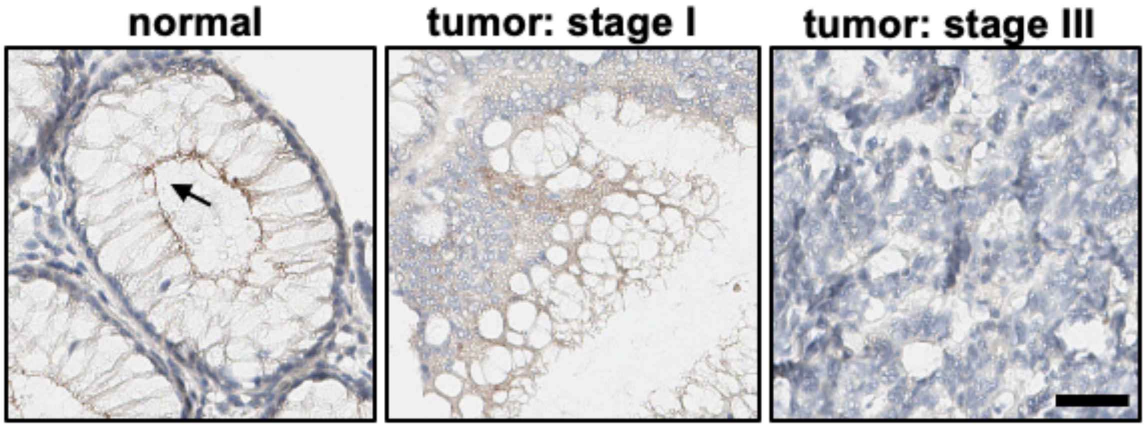


B

**PLEKHA7 status in colon cancer patient samples**

| | | normal | low - mislocalized | absent |
|---|---|---|---|---|
| | normal | 5 | 0 | 0 |
| **Stage:** | I | 0 | 3 | 0 |
| | IIA | 0 | 6 | 6 |
| | IIIA | 0 | 2 | 0 |
| | IIIB | 0 | 13 | 10 |

**Figure S2. PLEKHA7 dysregulation in colon cancer.** (**A**) Representative images and (**B**) assessment of PLEKHA7 immunohistochemistry in a cohort of commercially available colon normal and tumor patient tissues (n=45). PLEKHA7 is apical in normal tissues (see black arrow), but mis-localized or absent in all tumor samples. Scale bar: 20 μM.

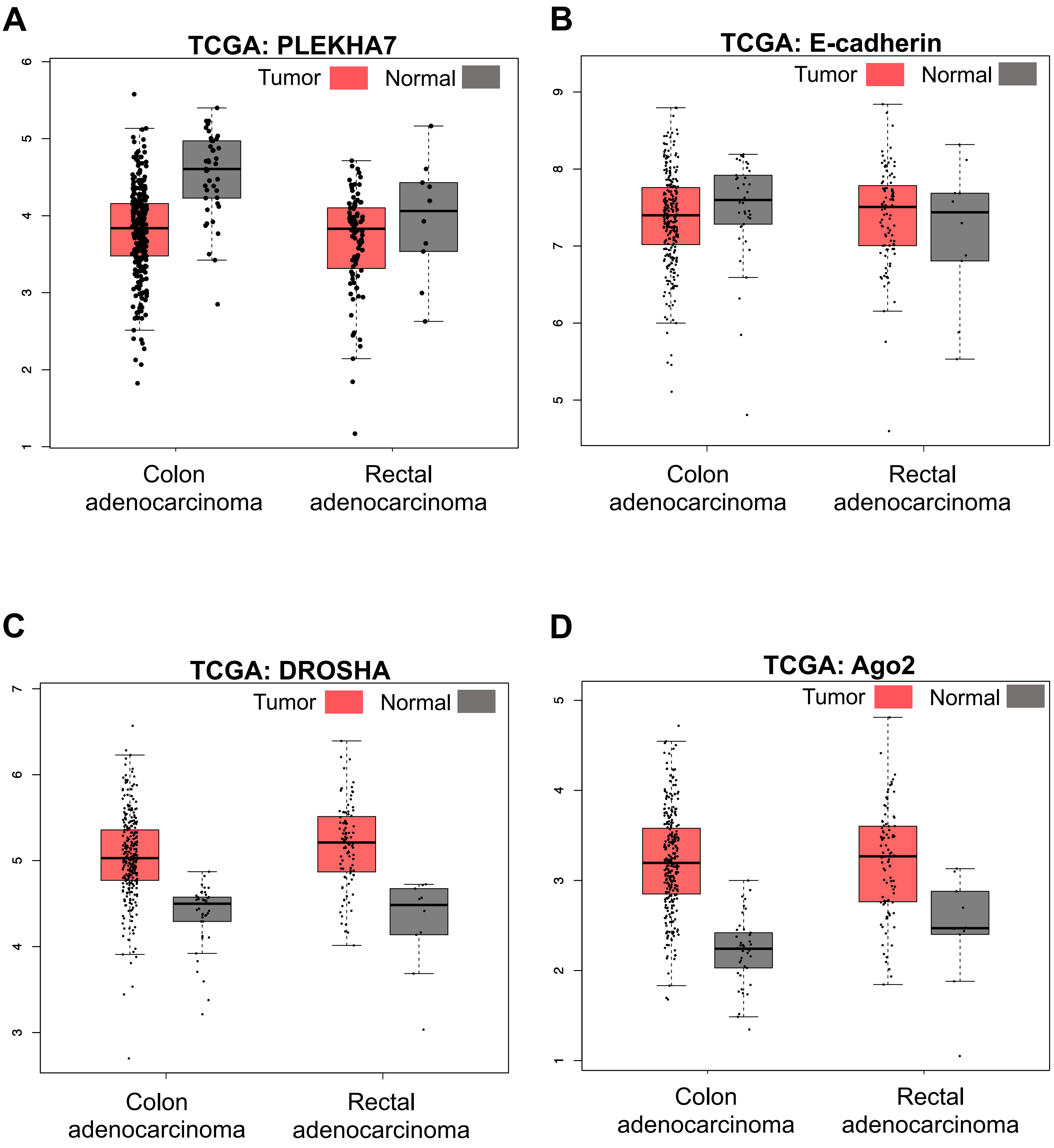


**Figure S3. TCGA expression data analysis.** Box plots depicting TCGA colon and rectal adenocarcinoma expression data for (**A**) PLEKHA7, (**B**) E-cadherin, (**C**), DROSHA, (**D**) Ago2. Analysis was performed using the online Gene Expression Profiling Interactive Analysis (GEPIA). Total number of tissues analyzed were: colon tumors, 275; colon normal tissues, 41; rectal tumors, 92; rectal normal tissues, 10. In all cases, p-value cutoff was 0.01.
